# Factors influencing patient willingness to participate in genetic research after a myocardial infarction

**DOI:** 10.1186/gm255

**Published:** 2011-06-15

**Authors:** David E Lanfear, Philip G Jones, Sharon Cresci, Fengming Tang, Saif S Rathore, John A Spertus

**Affiliations:** 1 Henry Ford Hospital, Heart and Vascular Institute, Detroit, Michigan, 48202, USA; 2 Mid-America Heart Inst, Kansas City, Missouri, 64134, USA; 3 Washington University in St Louis, Department of Medicine, Division of Cardiology, St Louis, Missouri, 63108, USA; 4 MD/PhD Program, Yale University School of Medicine, New Haven, Connecticut, 06510, USA

## Abstract

**Background:**

Achieving 'personalized medicine' requires enrolling representative cohorts into genetic studies, but patient self-selection may introduce bias. We sought to identify characteristics associated with genetic consent in a myocardial infarction (MI) registry.

**Methods:**

We assessed correlates of participation in the genetic sub-study of TRIUMPH, a prospective MI registry (n = 4,340) from 24 US hospitals between April 2005 and December 2008. Factors examined included extensive socio-demographics factors, clinical variables, and study site. Predictors of consent were identified using hierarchical modified Poisson regression, adjusting for study site. Variation in consent rates across hospitals were quantified by the median rate ratio (MRR).

**Results:**

Most subjects consented to donation of their genetic material (n = 3,484; 80%). Participation rates varied greatly between sites, from 40% to 100%. After adjustment for confounding factors, the MRR for hospital was 1.22 (95% confidence interval (CI) 1.11 to 1.29). The only patient-level factors associated with consent were race (RR 0.93 for African Americans versus whites, 95% CI 0.88 to 0.99) and body mass index (RR 1.03 for BMI ≥ 25, 95% CI 1.01 to 1.06).

**Conclusion:**

Among patients with an MI there were notable differences in genetic consent by study site, but little association with patient-level factors. This suggests that variation in the way information is presented during recruitment, or other site factors, strongly influence patients' decision to participate in genetic studies.

## Background

As genetic research becomes more common and genetic factors are studied as a means for improving risk stratification and treatment, it is essential that participating subjects are representative of the general population of patients from which they are recruited. However, genetic research often attains lower participation rates compared with non-genetic studies [1]. Failure to recruit eligible subjects may also introduce selection biases into genetic studies, potentially jeopardizing both internal and external validity. Existing studies addressing this issue have revealed participation rates for genetic studies ranging from 21% to 99% [2-5]. This variability depends on many factors, including the disease under study [6], circumstances in which the patient is recruited [5], as well as a variety of patient characteristics that may impact patients' willingness to participate, including race [7,8], education [9,10], and gender [3,7,8,11].

The existing literature has limited data regarding the genetic participation of patients with acute illnesses, which are required to study common cardiovascular diseases such as myocardial infarction (MI). First, some of the larger published studies are based upon opinion surveys (that is, asking whether the subject would be willing to participate in a theoretical genetic study) [2,10,12]. While these are important to help illuminate subjects' decision-making processes, subjects considering actual sample donation may behave differently when faced with the reality of undergoing blood/tissue collection, the potential risk of a confidentiality breach, or other real or perceived consequences of genetic analyses. Among studies that did involve actual donation and storage of samples, few have included large numbers of patients enrolled during the course of acute illness, which may also affect participation rates. For example, dissimilar participation rates have been reported for population-based studies compared to hospital-based cohorts [5]. Further clouding the literature is that most genetic studies have not adequately described populations from which samples were recruited, precluding an assessment of participation bias and potentially affecting internal and external validity [5]. The only study we are aware of examining genetic participation rates in an MI registry found significant clinical differences between participants and non-participants [13], calling into question the external validity of this study. A better understanding of the variability in patients' willingness to participate in genetic studies of acute cardiovascular disease is needed to assess for selection biases and to identify opportunities to improve participation and optimize the generalizability of such studies.

To address this existing knowledge gap, we examined characteristics associated with participation in a genetic sub-study within a large multi-center registry of MI patients. TRIUMPH (Translational Research Investigating disparities in Myocardial infarction Patients' Health status) is a multi-center study of MI patients at 24 US centers spread across the country and representing urban, suburban, academic, and community hospitals. A principal goal of this study was to assess genetic and pharmacogenomic factors associated with post-MI outcomes. Each enrolled patient was invited, but not required, to contribute DNA for genetic study. Participating hospitals were from diverse regions, including academic and community centers, a broad spectrum of economic and racial populations, as well as urban and rural locations. As such, TRIUMPH provided an ideal opportunity to examine patient factors associated with participation in genetic studies. Specifically, we sought to identify factors associated with genetic study participation, and to examine any differences between participants and those who chose not to donate their genetic material, in the hopes that these insights could improve recruitment in future studies.

## Materials and methods

### Participants

All data and analyses presented here are part of the TRIUMPH study, a prospective registry of patients with acute MI from 24 hospitals across the United States (listed in Acknowledgements). The study was Institutional Review Board approved at all participating sites, and written informed consent was obtained from all participants. All patients who entered the registry were also offered participation in the genetic sub-study; however, this was not mandatory (that is, patients could participate in the registry without contributing DNA). A Federal Certificate of Confidentiality was obtained to further protect the confidentiality of patients' information and this was disclosed to patients in the informed consent document. Patients were enrolled from April 2005 to December 2008.

### Data collection

For each patient in the parent study, detailed clinical and treatment characteristics were collected by chart abstraction and interview. Trained data collectors at each site participated in the acquisition of requisite data. Factors examined for association with genetic study participation included the socio-demographic, financial, social support, medical literacy, health status, depressive symptoms, clinical variables listed in Table 1 and enrollment site. All psychosocial and health status characteristics were quantified using standardized instruments, as previously described for the PREMIER study [14].

**Table 1:** Patient characteristics in genetic sub-study participants versus non-participants

	Consented to use of DNA		
		
	Yes (n = 3,484)	No (n = 856)	*P*-value
**Demographics**			
Age	58.9 ± 12.2	59.8 ± 12.7	0.038
White/Caucasian race	2,342 (67.4%)	573 (67.3%)	0.981
Male	2,347 (67.4%)	551 (64.4)	0.095
Language			0.293
English	3,353 (98.4%)	823 (97.9%)	
Spanish	55 (1.6%)	18 (2.1%)	
Missing	76	15	
Ethnicity			0.870
Hispanic/latino	217 (6.4%)	54 (6.6%)	
Non-hispanic/latino	3,175 (93.6%)	770 (93.4%)	
Unknown	92	32	
			
Low social support	612 (18.3%)	109 (13.1%)	< 0.001
Missing	133	23	
REALM-R score ≤ 6	844 (28.5%)	170 (28.1%)	0.823
NA, missing, or unknown	423	250	
			
**Socio-economic status**			
Completed high school	2,764 (79.8%)	656 (76.9%)	0.058
History of avoiding medical care due to cost	904 (26.5%)	184 (21.7%)	0.004
End-of-month financial situation			<0.001
Some money left over	1,380 (40.4%)	397 (47.3%)	
Just enough to make ends meet	1,297 (37.9%)	295 (35.1%)	
Not enough to make ends meet	741 (21.7%)	148 (17.6%)	
			
**Medical history**			
BMI	29.8 ± 10.2	29.0 ± 6.5	0.025
Chronic heart failure	302 (8.7%)	70 (8.2%)	0.646
Dyslipidemia	1,721 (49.4%)	407 (47.5%)	0.332
Hypertension	2,318 (66.5%)	575 (67.2%)	0.722
Prior MI	710 (20.4%)	202 (23.6%)	0.038
Cancer	250 (7.2%)	62 (7.2%)	0.946
Diabetes	1,068 (30.7%)	268 (31.3%)	0.710
			
**Presentation**			
Final MI diagnosis			0.568
STEMI	1,475 (42.3%)	383 (44.7%)	
NSTEMI	1,979 (56.8%)	465 (54.3%)	
BBB/uncertain type	7 (0.2%)	2 (0.2%)	
Patient not diagnosed with MI	23 (0.7%)	6 (0.7%)	
Peak troponin	29.3 ± 76.6	25.6 ± 58.1	0.187
			
**Medications (arrival and discharge)**			
Aspirin on arrival	1,431 (41.1%)	353 (41.2%)	0.930
Beta blocker at DC	3,109 (89.7%)	776 (91.4%)	0.132
Thienopyridine on Arrival	433 (12.4%)	113 (13.2%)	0.541
Statin at DC	3,030 (87.4%)	744 (87.6%)	0.852
			
**Processes of care**			
In-hospital cardiac catheterization	3,222 (92.5%)	777 (90.8%)	0.096
In-hospital revascularization	2,498 (71.7%)	618 (72.2%)	0.772
Enrolled in other study	326 (9.4%)	70 (8.2%)	0.283
Length of stay	5.6 ± 6.4	6.0 ± 8.90	0.207
			
**Health Status**			
SAQ Quality of Life score	62.2 ± 23.6	67.5 ± 23.3	< 0.001
SAQ Angina Stability score	43.8 ± 21.8	47.1 ± 20.6	< 0.001
SAQ Physical Limitation score	85.0 ± 22.6	88.4 ± 19.6	< 0.001
SF-12v2 Mental Component score	49.6 ± 11.5	50.0 ± 11.6	0.411
SF-12v2 Physical Component score	42.0 ± 12.4	42.8 ± 12.5	0.094
PHQ-9 depression severity			<0.001
Not clinically depressed	1,763 (54.4%)	539 (65.7%)	
Mild depression	831 (25.6%)	170 (20.7%)	
Moderate depression	376 (11.6%)	72 (8.8%)	
Moderately severe depression	191 (5.9%)	27 (3.3%)	
Severe depression	81 (2.5%)	12 (1.5%)	
Missing	242	36	
GRACE 6 m Mortality Risk score	100.0 ± 29.81	103.0 ± 31.1	0.008

Baseline patient characteristics are listed in the left-most column, with the quantities for those that participated in the genetic study, those that did not, and the *P*-value for difference between the two in the subsequent three columns. Categorical variables are shown as the number of subjects with that characteristic, followed by the proportion this represents (percentage) in parentheses. For variables that have subcategories, each subcategory and the number and proportion of subjects in that group is shown. Continuous variables are shown as the mean ± the standard deviation. Categorical variables were compared using chi-square or Fisher's exact test. Continuous variables were compared using Student's *t*-test. BBB, bundle branch block; BMI, body mass index; DC,; GRACE, Global Registry of Acute Coronary Events; NA, not applicable; NSTEMI, non-ST elevation myocardial infarction; PHQ, Patient Health Questionnaire; SAQ, Seattle Angina Questionaire; SF, Short Form; STEMI, ST elevation myocardial infarction.

All study staff underwent similar training at in-person meetings. Ongoing data collection issues were addressed through monthly conference calls. There were similar staffing ratios (per-recruited patient) across sites. Templates for informed consent documents and educational pamphlets about the study were provided and used or modified by each site.

### Statistical analyses

Patients were divided into two groups based on whether they consented to donate their genetic material (DNA) for storage and study, or not. Patient characteristics were compared using Chi-square tests for categorical variables and *t*-tests for continuous ones. The likelihood of consent was modeled using hierarchical regression that included a random effect for hospital. Because the consent rate was high, we estimated rate ratios (RRs) directly (that is, instead of estimating odds ratios) by using a modified Poisson regression model with robust standard errors [15]. For multivariable models, we initially included characteristics thought *a priori *to be associated with participating in the genetic sub-study. These included age, race, gender, education, finances, social support, symptom severity, and hospital. In order to assess for potentially important patient characteristics, we also included the characteristics from Table 1 that showed univariate association with participation (*P *< 0.05) in the multivariable models. Variation in consent rates between hospitals was quantified by the median rate ratio (MRR), which estimates the average relative difference in likelihood of two hypothetical patients, with identical covariates, consenting if enrolled at two different hospitals. Site participation rates are shown as smoothed estimates, which are a weighted average of the hospital's individual rate and the overall rate for the entire cohort, where the weight given to an individual hospital is roughly proportional to their sample size. Smoothing was used in order to take into account the fact that some hospitals have small sample sizes and thus more uncertainty around their true rate.

Approximately 16.1% of patients had missing covariate data (13.8% were missing one value, 1.8% were missing two values, and 0.5% were missing three or more values; the highest missing rate for any single variable (Patient Health Questionnaire (PHQ) depression score) was 6.4%. Missing covariate data were imputed with multiple imputation using IVEwareE [16]. All analyses were performed in SAS version 9.1.3 (SAS Institute, Cary, North Carolina, USA), and R, version 2.7.0 (Foundation for Statistical Computing, Vienna, Austria).

## Results

**Figure 1:**
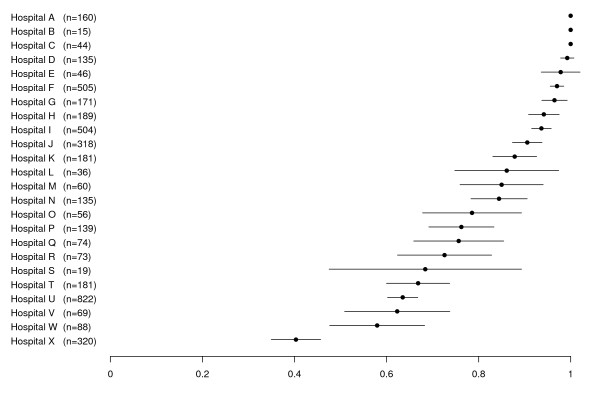
**Genetic consent rates by hospital**. Each hospital is labeled by letters A to H (vertical axis). Each dot and line represents the proportion of subjects at the site that consented to genetic sub-study enrollment. The central dot shows the point estimate of the site rate (percentage) generated from the random effects models. The lines extending from the dot represent the 95% confidence interval.

A total of 4,340 patients were enrolled in the study. Of these, 3,484 (80%) consented to donate their DNA for study. Clinical and socio-demographic characteristics among genetic sub-study participants versus non-participants are summarized in Table 1. Several socio-demographic factors differed between participants and non-participants in unadjusted analyses, including measures of social support, literacy, education, financial hardship, and smoking status. Among clinical variables, health status, body mass index (BMI), history of MI, history of stroke, and receiving beta-blockers on arrival each had univariate associations with genetic consent. The genetic participation rate varied across enrolling sites, ranging from 40% to 100%. Smoothed estimates of site participation rates derived from the random effects model are shown in Figure 1.

**Figure 2:**
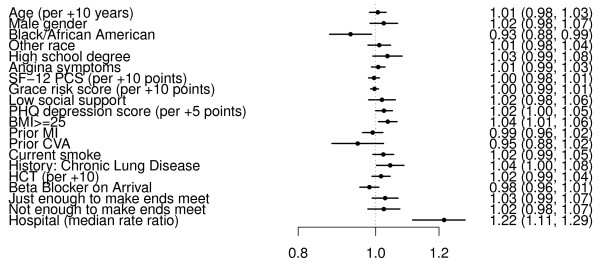
**Multivariable model of participation in genetic sub-study**. Variables included in the model are shown along the vertical axis. The strength of effect is shown along the horizontal axis with the vertical dotted line demarking a rate ratio of 1 (that is, no effect); estimates to the right (that is, > 1) are associated with greater likelihood of genetic consent while those to the left (that is, < 1) indicate association with reduced likelihood of genetic consent. Each dot and line represents the point estimate of the effect of that variable in the model, while the line shows the 95% confidence interval. CVA, Cerebral Vascular Accident; HCT, hematocrit; PHQ, Patient Health Questionnaire; SF-12 PCS, short form 12 physical component score.

A multivariable modified Poisson model was then constructed to test for factors associated with consenting to genetic testing (Figure 2). The only factors independently associated with participation were African American race, enrollment site, and BMI. African American race was associated with a 7% lower rate of consenting to genetic study compared with white patients (RR 0.93; 95% confidence interval (CI) 0.88 to 0.99). Higher BMI (≥ 25) was marginally associated with a slightly higher participation with RR of 1.03 (95% CI 1.01 to 1.06). Several other factors were of borderline significance, including PHQ-9 score (RR 1.02 for every 5 points; 95% CI 1.00 to 1.05) and chronic lung disease (RR 1.04; 95% CI 1.00 to 1.08). By far, the strongest factor associated with participating in the genetic study was enrollment site. The MRR was 1.22 (95% CI 1.11 to 1.29), suggesting that an identical patient presenting at one hospital would, on average, have a nearly 1 in 4 greater likelihood of participating in a genetic study than if that same patient had presented to a different TRIUMPH hospital.

## Discussion

We sought to define characteristics associated with participation in a genetic sub-study of a large acute MI registry. We found that the vast majority of patients chose to participate in genetic testing (around 80%), with few differences between those who did and did not agree to donate DNA. Although we found race to be mildly associated with patients' willingness to participate in genetic studies, other factors such as gender and education level were not. Most importantly, the strongest predictor of participation in the genetic sub-study was hospital site, with wide variability seen in rates across sites.

Reduced genetic participation among racial minorities is a particularly critical issue since racial disparities in health outcomes are high-priority research topics, and the genetic versus non-genetic components of health disparities need to be better elucidated. Higher rates of participation in genetic studies among white patients, as compared with African Americans, have been previously described [4,7,8]. A lower likelihood of African American participation in medical research generally has also been well described, with lack of trust or confidence in the researchers being one important factor [17]. Similarly, trust is one of the most often cited mediating factors for participation in genetic studies [2], and this is also the case in studies specifically focusing upon racial differences in genetic research; patient concerns about confidentiality were a consistent reason for choosing not to participate [12,18]. In our study, African Americans were 7% less likely to participate than whites, a modest difference in participation rates. While further qualitative studies may help illuminate the mechanism, awareness of this potential selection bias is important during study enrollment so that under-representation of racial minorities can be minimized. Making every effort to establish trust and rapport with subjects, as well as confidence in the research team and their confidentiality protections, may help reduce refusal rates.

To our knowledge, the association of genetic consent with BMI has not been previously reported, and the magnitude of the association is of questionable clinical significance. Given the number of possible predictors included in this study, this association may be spurious. Confirmation of this finding in an independent cohort is needed and, if consistent findings are observed, then qualitative research could be used to better understand the potential mechanism of this association. In contrast to previous studies, our data did not show any other patient level characteristics to be significantly associated with patients' willingness to consent to genetic testing.

Some additional aspects of our data are worth noting. First, our study examined acutely ill hospitalized patients, while most previous studies were outpatient or population-based. We found rates of participation roughly similar to previous studies of patients that had already consented to non-genetic research [4,19,20]. The only other published genetic MI registry addressing participation rates [13], identified clinical selection biases, but these were not confirmed in ours. In contrast, our data demonstrated that patients consenting to genetic participation were overall quite similar to those who chose not to participate across a wide range of clinical factors. This difference may be due to the fact that ours is a multi-center cohort, as opposed to the single-center experience of the previous study. Given the importance of recruitment site in our study, there may have been unique characteristics of that site that influenced their findings. Nevertheless, it is critically important that genetic association studies explicitly quantify potential selection biases of the participating cohort compared with the parent population to whom the conclusions will be applied. The similarity of our genetic versus non-genetic patients supports the external validity of the future genetic analyses planned for these data.

Most importantly, we were able to clearly identify that site of recruitment was the most important factor associated with participation. While the mechanism can not be stated with certainty, this most likely reflects variations between centers in the presentation style of individual study coordinators, their motivation to recruit into the genetic study, ability to establish rapport and trust, or their ability to provide complete information to patients' satisfaction and comfort. If this is true, the marked variation across sites indicates an important opportunity, through better training and standardization, to improve enrollment processes in future studies. Ensuring high-quality and consistent consent processes should reduce variability in consent rates and may also provide overall enhanced participation in genetic association studies. This is highly desirable in order to minimize the potential for bias and enhance generalizability. Although specific training regarding genetic enrollment was done at the beginning of our study, changes in study coordinators and shifts in their responsibilities may have limited the effectiveness of the initial standardization for DNA acquisition across sites. Moreover, testing, through role-playing, coordinators' skills in obtaining informed consent are important steps for future studies to consider. We further suggest that future studies provide ongoing assessments of the rates of genetic consents at each center to rapidly identify differences between site participation rates so that proactive education of site coordinators can occur throughout the study. These data also underscore the importance of close collaborations between investigators, coordinators, Institutional Review Boards and others involved in genetic studies to optimize communication with subjects, assess their comprehension, and to provide strong protections (for example, confidentiality) that can maximize patient comfort with, and participation in, genetic research.

Our findings should be interpreted in the context of the following potential limitations. First, we can not completely exclude the possibility that unidentified variation in patient characteristics between sites may have led to residual confounding of the observed differences in participation rates. Specifically, there could theoretically be regional differences in patient attitudes towards genetic study that influence participation decisions that were not quantifiable from our extensive data collection, given that geographic region and enrollment site are highly correlated. Second, our data do not identify the mechanism underlying our observed associations, which would require additional qualitative studies to better understand determinants of patient decision-making.

## Conclusions

Our multi-center study was able to engage 80% of patients to participate in genetic research at the time of their acute MI. Genetic participants were clinically similar to those who chose not to donate their genetic material. African American patients, as compared with white patients, had a slightly lower rate of genetic participation, but no other patient-level factors, including gender and education, were significantly associated with consent. While BMI was statistically associated with participation rates, the magnitude of the effect was small and this association has not been previously observed to our knowledge. Most importantly, the strongest factor associated with genetic consent was enrollment site. This suggests that differences in how study personnel interact with patients are a key determinant of their willingness to participate, and should be prospectively monitored in future studies to maximize participation rates in genetic investigations.

## Abbreviations

BMI: body mass index; CI: confidence interval; MI: myocardial infarction; MRR: median rate ratio; PHQ: Patient Health Questionnaire; RR: rate ratio; TRIUMPH: Translational Research Investigating disparities in Myocardial infarction Patients' Health status.

## Competing interests

The authors declare that they have no competing interests.

## Authors' contributions

DEL contributed to the study conception and design, data analysis, drafted the manuscript, and approves of the final manuscript. PGJ contributed to the acquisition of data, data analysis, critically revising the manuscript, and approves of the final manuscript. SC contributed to the data analysis and interpretation, critically revising the manuscript, and approves of the final manuscript. FT contributed to the acquisition of data, data analysis, critically revising the manuscript, and approves of the final manuscript. SSR contributed to the data analysis and interpretation, critically revising the manuscript, and approves of the final manuscript. JAS contributed to the study design, data analysis, drafting of the manuscript, and approves of the final manuscript.
